# Attenuation of stripe artifacts in optical coherence tomography images through wavelet-FFT filtering

**DOI:** 10.1364/BOE.10.004179

**Published:** 2019-07-24

**Authors:** Robert Byers, Stephen Matcher

**Affiliations:** 1 Dept. of Infection & Immunity & Cardiovascular Disease, Univ. of Sheffield, Beech Hill Road, Sheffield, S10 2RX, UK; 2 Dept. of Electronic and Electrical Engineering, Univ. of Sheffield, Broad Lane, Sheffield, S3 7HQ, UK

## Abstract

The use of polarization-maintaining (PM) fibers for polarization-sensitive optical coherence tomography (PS-OCT) can result in numerous image artifacts which degrade the reliability of birefringence measurements. Similar artifacts can also arise in conventional OCT, due to stray reflections from optical surfaces, a problem which is increasing in tandem with the steady rise in source coherence lengths. Here, a recently presented wavelet-FFT filter[Opt. Express 17(10), 8567 (2009).1943419110.1364/oe.17.008567] is combined with surface flattening displacement fields in order to suppress ghost artifacts following either a duplicate or inverse profile to that of the sample surface. In addition, horizontal coherence stripes originating from Fresnel reflections of optical components are suppressed in order to facilitate accurate surface detection. The result is an improved visualization of the phase-retardance profile within tissue, which may improve the reliability of curve-fitting methods for localized birefringence estimation. While the results are presented with a focus towards PS-OCT, the filtering method can also be applied to the removal of stray reflection artifacts in conventional OCT images.

## 1. Introduction

Optical coherence tomography (OCT) is a non-invasive optical modality, capable of producing high-resolution cross-sectional images of tissue structure to a depth of a few millimeters [2]. One downside of regular, intensity-based OCT is a lack of tissue specific contrast, which often makes it challenging to discern between regions of tissue which exhibit similar refractive indices. As a result, numerous functional extensions to OCT have been developed, each aiming to exploit unique properties of the light and tissue in order to extract additional contrast. Such extensions include: angiographic OCT [3–6], spectroscopic OCT [7], OCT elastography [8] and polarization-sensitive OCT (PS-OCT) [9].

PS-OCT utilizes measurements of the polarimetric information carried by transverse light waves to measure properties such as the phase retardation (δ) [10,11] and fast birefringent axis orientation (ϑ) [12] within a sample. It has found extensive use within biological fields, notably within dermatology [13], opthalmology [14], dentistry [15] and for the enhanced study of tissues such as bone, tendon, ligament and cartilage [16]. Recently the use of single-mode (SM) fiber to construct PSOCT systems has increased in popularity [17–19], however PM fiber is highly stable in its birefringence properties and allows the relatively simple “single input state” method to be applied to fiber-based systems [20]. Indeed, many recently developed PS-OCT systems make use of polarization-maintaining (PM) fibers [20–22] which have less systematic errors and are often more clinically applicable when compared to systems utilizing bulk optics [23]. However such systems are not without disadvantage; the polarization-maintaining property of the fiber is achieved by inducing an intentional linear birefringence within the fiber, such that the two polarization modes (Horizontal and Vertical) propagate at distinct, and significantly different phase velocities. Effectively, beam propagation is slowed along one axis of the elliptic fiber core with respect to the other [21]. Slight imperfections in the splice angles and alignment of fiber connectors can result in undesirable cross-coupling between the two axis [21,22], which when detected and processed will result in vertically offset “ghost” copies of the OCT signal. While such “ghost” images are typically much lower in intensity that the original image, their bright surface profiles have sufficient intensity to noticeably degrade the underlying image. A hardware based method of removing such artifacts involves displacing them out of the OCT field-of-view through usage of long PM fiber segments [22]. Here, we propose a post-processing based algorithm based around wavelet-FFT filtering [1] which aims to attenuate the surface ghost reflections such that artifact-free images of the phase-retardance profile can be obtained, without the requirement for any hardware based compensation or modification.

Fig. 1

**Fig. 1:**
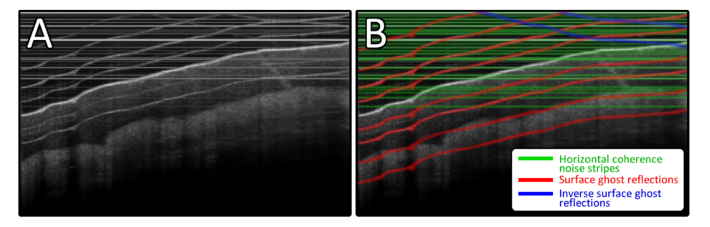
A) A structural PS-OCT scan of the palm showing the three common image artifacts. B) Artifacts of each type have been overlaid with a specific color: Green shows horizontal coherence noise stripes, red shows surface ghost reflections and blue shows inverted surface ghost reflections.

. illustrates the primary artifact types which this algorithm aims to address. All three artifacts are common occurrences within PM-fiber based PS-OCT images, with horizontal coherence noise stripes also affecting conventional OCT:

1. **Horizontal coherence noise stripes** – Uniform horizontal lines spanning the entire width of the image. Derived from Fresnel reflections from optical components within the system.
2. **Surface ghost reflections** – Cross-coupling between the orthogonal polarization modes within the PM-fibers causes vertically offset copies of the OCT image to be present both above and below the sample. While these “ghost” images generally have a much lower signal intensity, their surface profiles are sufficiently bright such that the underlying image is significantly degraded. Thus the focus here is on the removal of the bright surface profile, rather than the entire “ghost” image.
3. **Inverted surface ghost reflections** – Complex-conjugate derived “mirror-artifacts” which arise from ghost artifacts which are positioned above the zero optical-path-difference (OPD) point of the system. These become more problematic the closer the sample surface is to the location of the zero OPD.

## 2. Methods

### 2.1 PS-OCT system

**Fig. 2:**
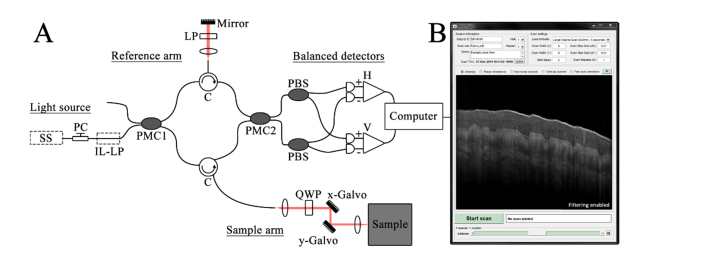
A) Schematic diagram of the PM-fiber based PSOCT system. SS: Swept-source light source, PC: polarization controller, IL-LP: in-line linear polarizer, PMC1/2: polarization-maintaining couplers, QWP: quarter waveplate, PBS: polarization beamsplitter, H/V: Balanced photo-detectors which detect the horizontal and vertically polarized light respectively. B) MATLAB general user interface for acquisition and live processing of the data.

A PM fiber based PS-OCT system was used to collect images of tissue for processing. The system follows the scheme originally reported by Al-Quasi *et al* [22] and has been detailed fully in a previous publication [24]. Figure 2

shows a schematic of the system. The light source used was a commercially available swept-source laser (HSL-2000-10-MDL, Santec Japan) which has a center wavelength of 1315nm, a sweep range of 157nm and a full width at half maximum (FWHM) of 128nm. The measured axial resolution (in-air) of the system was approximately 10μm and the laser sweeps at a rate of 10 kHz. Circularly polarized light is directed to the sample via a Panda PM-fiber Mach-Zehnder interferometer. Backscattered light from the sample is directed towards two balanced detection channels (1817-FC, New Focus, US) which measure the interferometric signal corresponding to the horizontal and vertical orthogonal polarization states of the light. In linearly birefringent materials, light with a linear polarization which is aligned parallel to the optic axis of the material experiences a different index of refraction (and thus propagation speed) compared to light with a linear polarization aligned perpendicular to the optical axis. Thus birefringence within the sample causes a phase-retardance between the complex amplitudes of each orthogonal polarization channel, which is detected by considering the evolution of the ratio between channels as a function of tissue depth. A MATLAB (R2015b – MathWorks) general user interface was designed to facilitate both data acquisition and live processing of the images with the algorithm discussed herein.

### 2.2 Wavelet-FFT filtering

Horizontal or vertical line or “stripe” artifacts are a common occurrence within many imaging modalities, including OCT. As a uniform stripe has a very high spatial frequency bandwidth along its width but a minimal frequency bandwidth along its length direction, a common method of removing such artifacts involves the use of the frequency domain. Thus, a basic implementation of a de-striping algorithm may simply calculate the 2D FFT $F(u_{x},u_{y})$ of an image f(x,y), then attenuate frequencies along the axis which is perpendicular to the line while maintaining the coefficients in the neighborhood of the origin. Either: $\{F|u_{x}=0,u_{y}\neq 0\}=0$ for horizontal lines or $\{F|u_{x}\neq 0,u_{y}=0\}=0$ for vertical lines. The filtered image can then be recovered using the inverse FFT operation. One limitation of this approach is that image detail coefficients are prone to being deleted alongside the detrimental stripes, causing a loss of underlying image information.

**Fig. 3:**
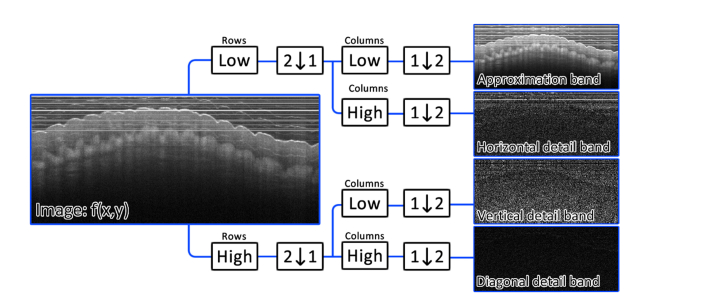
A single-level wavelet decomposition of a PS-OCT structural image. Low and High refer to a Daubechies decomposition low-pass filter and highpass filter respectively, the image data is convolved with these filters along either the columns or rows. **1↓2** refers to a down-sampling of the rows while **2↓1** refers to a down-sampling of the columns.

An improved method of filtering stripes from images was recently proposed by Münch *et al* [1]. They demonstrated that by first wavelet filtering the image, such that the approximation, diagonal detail, vertical detail and horizontal detail components of an image are reversibly condensed into separate bands, the aforementioned FFT filtering can be performed only on the relevant artifact corrupted band (horizontal in this case), thereby improving preservation of the underlying image information when compared to the purely FFT based method [1]. Figure 3

shows how this wavelet decomposition is performed.

The image f(x,y) is fed through a bank of low and high pass filters which operate along either the rows or columns of the image. Each time the image is filtered, it is essentially down-sampled by a factor of two by the filter. Low pass filtering both the rows and columns captures the low frequency approximation information of the image. Low pass filtering the rows but high pass filtering the columns captures horizontal detail information, due to horizontal lines having a high frequency along the columns and a low frequency along the rows. Similarly, vertical detail information is extracted by high pass filtering the rows and low pass filtering the columns. Lastly diagonal information is extracted by high pass filtering both the rows and columns. This process can be repeated on the approximation band multiple times in order to extract lines which were wider than 1-pixel in the original image (Lines become a factor of 2 “thinner” between each successive wavelet decomposition due to the down-sampling). The aforementioned FFT filter can then be applied to only the relevant artifact corrupted band (Horizontal detail band here) and the filtered image repackaged using inverse wavelet transformations. This combined “wavelet-FFT” filter itself contains numerous variables:

1. The decomposition level (l) is the number of successive approximation bands which are wavelet filtered, a higher level will filter wider lines from the image.
2. Sigma (σ) defines the width of FFT coefficients which are damped within each horizontal detail band. A larger value of sigma will filter increasingly “imperfect” lines from the image, for example lines which are not uniform along their length.
3. The type of wavelet used (e.g Haar, Daubechies 2, Daubechies 20).
4. The decomposition bands which are filtered, filtering specific bands would remove lines of a certain size range corresponding to the bands filtered.

**Fig. 4:**
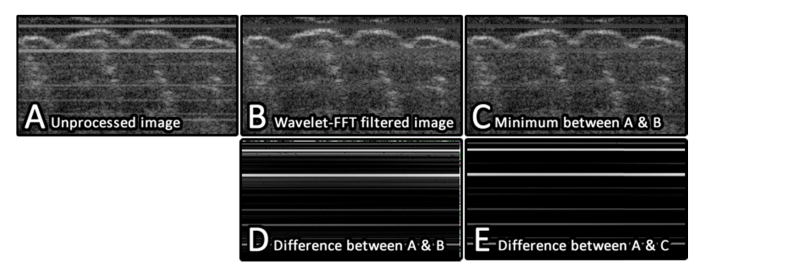
A) A small section of a PS-OCT reflectivity image which is contaminated with horizontal stripe artifacts. B) The image following wavelet-FFT filtering, the filtered lines have “blurred” out into the surrounding signal. C) The result of taking the minimum value between A and B. D) Absolute difference image between A and B. Bright regions show where the image has changed. There is a visible “blurring” around each of the line artifacts which indicates that signal previously not corrupted by artifacts has been affected. E) Absolute difference image between A and C. Following the application of the minimum operation, the change in signal intensity is restricted to the location of the horizontal artifacts only.

For this work, the wavelet-FFT filter parameters were optimized (See Sec. 3.1) with: l=4, σ=10 and a Daubechies 20 wavelet being selected as they offered a good balance between performance and processing speed. All horizontal detail bands were filtered in order to remove horizontal lines within the images. These parameters provided good results across a range of PS-OCT data sets, although improved results could potentially be obtained through optimization of the filter parameters for specific image cases. In addition, as the artifacts affecting the PS-OCT horizontal and vertical amplitude images are additive, they always appear *brighter* than the underlying true signal. Thus, a simple method of preventing the lines from “blurring” across previously unaffected signal [25] is to calculate the minimum value between the pre- and post-filtering images. This additional step was applied following each application of the wavelet-FFT filter. The benefits of this minimum filtering step are illustrated in Fig. 4

below.

### 2.3 Artifact removal algorithm

**Fig. 5:**
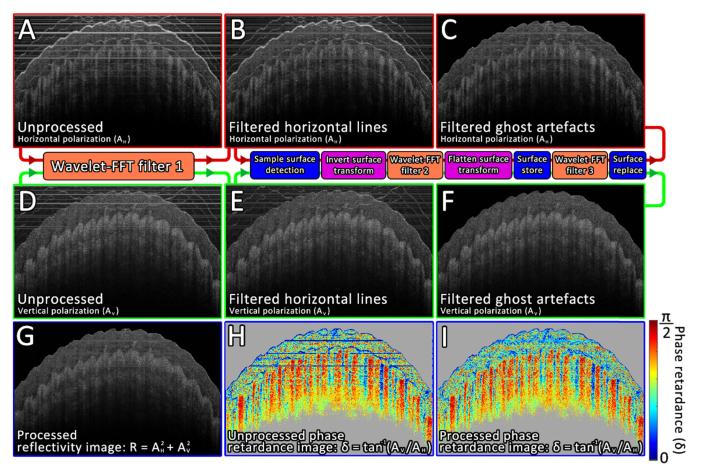
The processing pipeline for PS-OCT artifact reduction. A) Unprocessed PS-OCT scan of a fingertip showing horizontally polarized signal ($A_{H}$). B) $A_{H}$ following the first application of the wavelet-FFT filter, which aims to attenuate horizontal coherence noise stripes. C) $A_{H}$ following the second and third applications of the wavelet-FFT filter, which aim to attenuate both regular and inverted surface ghost reflections. Signal located above the detected skin surface has been set to zero. D-F) Same as A-C but showing the vertically polarized signal ($A_{V}$). G) Processed sample reflectivity image calculated as: $R=A_{H}^{2}+A_{V}^{2}$. H) Unprocessed phase-retardance map calculated as: $\delta =\tan^{-1}(A_{V}/A_{H})$. I) Processed phase-retardance map.

In order to efficiently filter the three artifact types shown in Fig. 1 without significantly degrading the underlying image, a multi-step image processing pipeline was applied to PS-OCT images derived from both the horizontal and vertically polarized channels. This processing pipeline is illustrated in Fig. 5

and described below. Firstly, the combined wavelet-FFT filter described in Sec. 2.2 is applied to both the horizontal and vertical polarized images (Fig. 5A/D) in order to heavily suppress any horizontal coherence noise stripes which are present (Fig. 5B/E). The assumption at this point is that the sample surface within the image is not flat, otherwise it would also be removed by the filter. The next step involves detecting the sample surface, which is easier in the absence of the horizontal coherence noise stripes, however care must be taken to avoid erroneously detecting ghosted signal as the sample surface. The full sample surface detection process has been detailed previously [26], briefly this involves detecting the maximum signal intensity along each A-scan to generate a rough surface profile. This profile is then refined using a connectivity-enforcing path-finding algorithm. Once the surface profile is known, a displacement field (D) is defined as the pixel-mapping transformation which would flatten the sample surface. The inverse of this transformation ($D^{-1}$) is first applied, flattening any *inverted* surface ghost reflections within the image, such artifacts are then attenuated through a second application of the wavelet-FFT filter. The now inverted surface is then flattened through two applications of the displacement field (2D). While a third application of the wavelet-FFT filter at this point would indeed attenuate regular ghost artifacts within the image, it would also attenuate the true sample surface itself. To avoid this, the sample surface ± 5-pixels were stored prior to the wavelet-FFT filter being applied for a third time; the stored sample surface layer was then restored to its original location.

The resulting horizontal (Fig. 5C) and vertical (Fig. 5F) images were now filtered of artifacts and could be processed into sample reflectivity (Fig. 5G) and phase-retardance (Fig. 5I) images.

## 3. Results and discussion

### 3.1 Filter performance optimization

To optimize the performance of the filter, an OCT image acquired from a commercial system (VivoSight – Michelson Diagnostics Ltd) which did not present any noticeable image artifacts had artificial artifacts added to it. For this, horizontal coherence noise stripes were modelled as horizontal lines between 1 and 3 pixels in diameter with a random opacity of between 30 and 70%. Surface ghost reflections were simulated by offsetting copies of the B-scan by increments of 30-pixels in both vertical directions and setting the opacity randomly to between 10 and 90%. Inverse surface ghost reflections were produced in a similar manner, using an inverted B-scan. The opacity values were selected empirically based on the brightness of typical artifacts observed in real PS-OCT images. This artifact corrupted B-scan was then processed using the proposed filtering methodology before being compared with the original “clean” B-scan through calculation of the peak signal-to-noise ratio (PSNR). PSNR is a quality metric which is commonly used to evaluate image processing algorithms, providing the underlying image does not change between comparisons [27]. It is calculated as: $PSNR=10\;\log_{10}(\frac{255^{2}}{MSE})$ for the 8-bit OCT images, where MSE is the mean square error between the processed image and the original B-scan. To enable comparison primarily within the tissue, noise above the skin surface was set to zero for all PSNR calculations (Including the original B-scan). In ideal cases, where the mean square error trends towards zero, PSNR trends towards infinity, thus higher values generally mean the image more closely matches the “clean” original. Table 1 shows the result of calculating PSNR for images processed with a range of filter parameters.

**Table 1:** Optimization of the filter parameters (, and wavelet type)

				**Wavelet type**									
				Daubechies			Coiflets		Symlets		Fejer-Korovkin		Discrete Meyer
				5	20	40	1	5	5	20	4	22	-
**Decomposition level (l)**	1		σ = 1	29.70	29.58	29.53	29.88	29.58	29.83	29.54	30.41	29.55	29.55
			σ = 10	29.73	29.58	29.54	29.95	29.59	29.88	29.56	30.54	29.55	29.56
			σ = 100	29.89	29.66	29.59	30.22	29.68	30.02	29.62	30.93	29.60	29.60
	2		σ = 1	31.96	31.45	31.49	31.81	31.43	31.68	31.20	31.74	31.51	31.28
			σ = 10	32.01	31.45	31.50	31.97	31.46	31.75	31.22	32.08	31.53	31.29
			σ = 100	32.35	31.66	31.65	32.54	31.72	32.12	31.43	32.84	31.74	31.45
	3		σ = 1	34.12	34.36	34.29	34.17	34.16	34.38	34.23	34.45	34.46	34.23
			σ = 10	34.33	34.42	34.41	34.36	34.27	34.52	34.30	34.59	34.53	34.32
			σ = 100	34.65	34.81	34.79	34.59	34.68	34.86	34.69	34.61	34.87	34.69
	4		σ = 1	35.54	35.72	36.06	35.74	35.80	35.81	35.67	35.39	36.14	35.60
			σ = 10	35.79	36.25	**36.64**	35.34	36.32	35.75	36.27	34.22	36.49	36.26
			σ = 100	34.68	35.81	**36.64**	33.78	35.53	34.63	35.76	32.38	35.37	36.56
	5		σ = 1	35.32	34.05	34.09	35.66	34.49	35.54	34.16	33.87	34.11	34.71
			σ = 10	34.36	34.88	35.02	33.24	35.43	34.41	35.28	30.82	34.97	35.56
			σ = 100	31.64	33.29	34.66	30.27	33.35	31.82	33.64	28.76	32.67	35.28

**Fig. 6:**
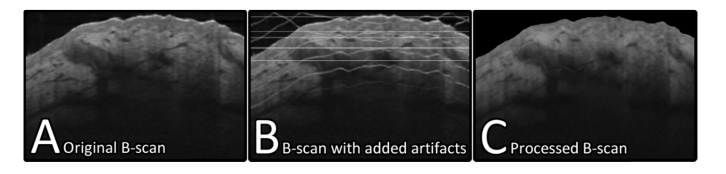
A) B-scan image of skin absent of any visible artifacts. B) The same image as A but artificial artifacts have been added to closely resemble a corrupted PS-OCT B-scan. C) The result of processing B with filter parameters: σ=10, l=4 and a Daubechies 40 wavelet.

The best performance was observed following filtering to 4 decomposition levels, using a Daubechies 40 wavelet with σ=10, this corresponded to a PSNR of 36.64. For reference, the PSNR of the unprocessed image was 27.19 thus the processed image more closely matches the original image. Figure 6

shows the visual difference between the original image, the artifact corrupted image, and the image processed using above parameters.

One aspect not discussed above is processing time, which varies as a function of the parameters. With this in mind, a Daubechies 20 wavelet was used for all other processed OCT images in this manuscript as it offers comparable PSNR (36.25) but speeds up processing by a factor of ~2x when compared to a Daubechies 40 wavelet. In addition, a limitation of the above analysis is the usage of only a single image. It is likely that the optimal value of parameters such as the decomposition level will vary depending on the spatial characteristics of the artifacts within the image (e.g artifact diameter).

### 3.2 Filter performance evaluation

**Fig. 7:**
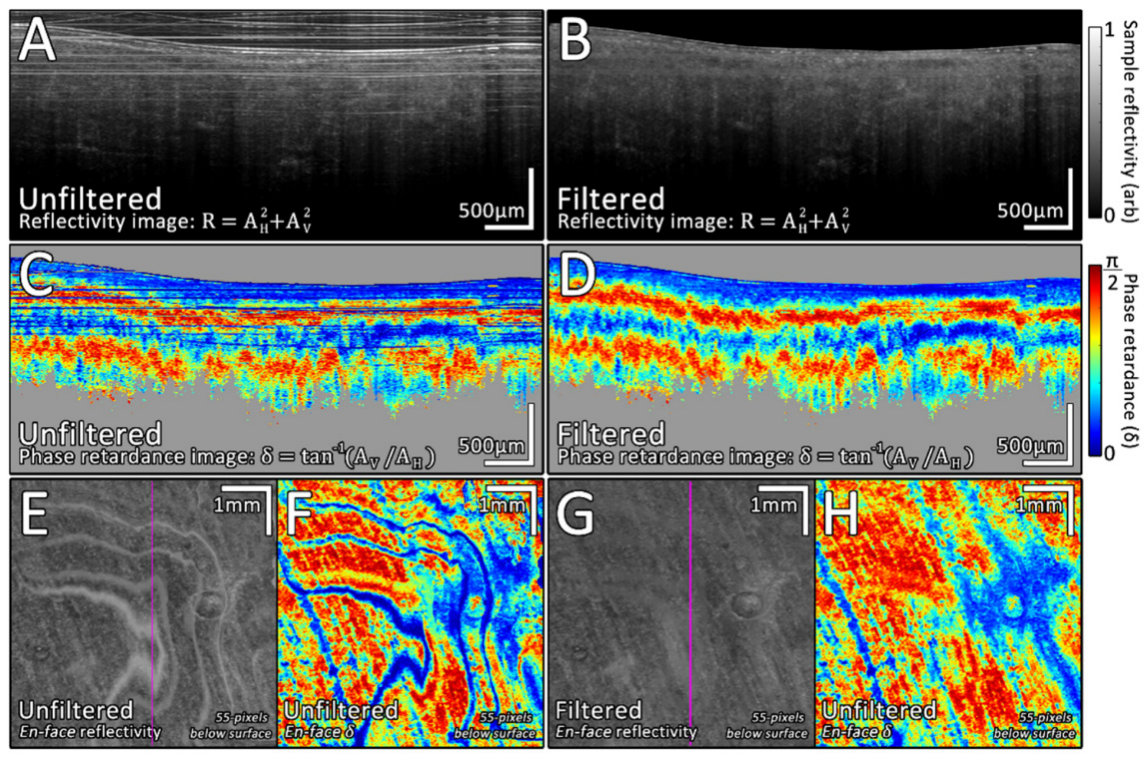
Results of processing PS-OCT images acquired from *ex-vivo* chicken breast tissue. A) Unfiltered sample reflectivity image. B) Filtered sample reflectivity image. Reflectivity calculated as: $R=A_{H}^{2}+A_{V}^{2}$. C) Unfiltered phase-retardance image, for comparison this has been masked using the same mask as that used for D. D) Filtered phase-retardance image, masked by excluding areas of low signal from the image (Defined as a processed sample reflectivity of less than 0.05). Phase retardance calculated as: $\delta =\tan^{-1}(A_{V}/A_{H})$. E) *En-face* view of the unfiltered reflectivity volume at a depth 55-pixels below the sample surface. F) *En-face* view of the unfiltered phase retardance volume at a depth 55-pixels below the sample surface. G) *En-face* view of the filtered reflectivity volume at a depth 55-pixels below the sample surface. H) *En-face* view of the filtered phase retardance volume at a depth 55-pixels below the sample surface. The pink vertical line on E/G shows the B-scan location used for the cross-sectional images.

To evaluate the performance of the algorithm using real PS-OCT data, a 6x6x2mm volume scan (Consisting of 1200x600x512 voxels) was collected from an *ex-vivo* sample of chicken breast tissue. Each slice of the volume was processed independently to generate both reflectivity and phase-retardance images following the methodology described in Sec 2.3. Figure 7

shows the results of this processing, to compare within the tissue only, the unfiltered phase-retardance images have been masked using the same mask as that used for the filtered phase-retardance images, this mask was generated as: $R_{Filtered}\left(x,y\right)>0.05$. The value of 0.05 was measured as the noise-floor by calculating the 95th percentile of pixel brightness in the bottom 1200x600x50 pixels of the data set.

Within the unfiltered sample reflectivity PS-OCT image (Fig. 7A), bright horizontal coherence lines and ghosting artifacts are visible both within the sample and above the sample surface. Such lines increase the difficulty of robust sample surface detection and reduce structural clarity within the image. In addition, the artifacts are clearly visible within the unfiltered phase-retardance image (Fig. 7C), typically manifesting themselves as blue lines with a phase-retardance close to 0. The reason the artifacts appear blue is due to them having a comparatively higher brightness within the horizontal channel than the vertical (Fig. 5A/D), thus: $\tan^{-1}(A_{V}/A_{H})\to 0$. Comparatively, both the filtered sample reflectivity image (Fig. 7B) and filtered phase retardance image (Fig. 7D) have visibly fewer artifacts than the unfiltered images, resulting in an improved visualization of the underlying tissue structure. The filtered images are not entirely artifact free, as small faint patches of ghost artifact can be seen towards the right side in addition to some faint banding at the upper left. From an *en-face* perspective across the entire volume, the artifacts are visible as wavy patches of bright reflectivity (Fig. 7E) or low phase retardance (Fig. 7F), due to them varying as a function of depth with respect to the sample surface. Following filtering, these wavy artifacts have been almost entirely attenuated (Fig. 7G/H) and are only visible as a faint blur in the reflectivity image.

Remaining artifacts which have persisted through the filtering process could potentially be addressed in two ways. Firstly, any minor imperfections in the sample surface detection will result in surface ghost reflections not aligning in a perfectly flat manner following the surface transformation process, resulting in non-uniform offset parameters in the frequency-domain. Thus, if further improvements can be made to accurately detect the sample surface, then the efficiency of filtering will likely increase. Secondly, further optimization of the wavelet-FFT filter parameters discussed in Sec. 2.2 could yield improved results, however the exact choice of parameters will likely depend on how far the various stripes affecting the images differ from ideal, 1-pixel wide horizontal lines. For example, increasingly wide line artifacts can be more effectively removed by increasing the decomposition level (l) of the filter at the expense of some degree of image information. A more efficient approach in terms of preserving the underlying image information, particularly for blurred lines such as those observed here, may be to specifically FFT filter the decomposition levels which correspond to the range of stripe widths within the image [1]. The entire filtering process, carries a processing overhead of ~1 second per 1200x512 pixel frame within MATLAB (Using a 3.5GHz i7 3770K CPU), thus this algorithm is unsuitable for high-speed real-time processing without further optimization; it is better suited for bulk offline processing of PS-OCT data sets following acquisition. In addition, although developed here specifically for filtering PM-fiber based PS-OCT images, the algorithm could be useful for cleaning regular OCT images that are contaminated by stray reflections from straight and/or curved surfaces.

## 4. Conclusions

In summary, the proposed algorithm provides a means of filtering parasitic optical artifacts from OCT and PS-OCT data sets. The algorithm functions through a 3-step “stripe-removal” process whereby line or stripe artifacts are attenuated within an image using a combined wavelet-FFT filter. The image is filtered in its regular, inverted surface and surface flattened states to remove the different artifact types. Filtered images contained visibly attenuated coherence noise stripes together with a reduction in the intensity of both regular and inverted surface ghost reflections, with little apparent degradation to the underlying structural image. Importantly, the algorithm functions without any hardware modification to the OCT system, and can be easily applied retroactively to previously acquired scans. In general, this method enables a clearer visualization of both the structural sample reflectivity image and the corresponding phase-retardance plot. Curve-fitting methods used to estimate local birefringence within the tissue are likely to be more robust and require lower amounts of smoothing in the absence of the artifacts.

Future work will focus on the optimization of the filter parameters for specific data sets, allowing for more complete filtering of any present artifacts.

## References

[1] Münch B. Trtik P. Marone F. Stampanoni M., “Stripe and ring artifact removal with combined wavelet--Fourier filtering,” Opt. Express 17(10), 8567–8591 (2009). 10.1364/OE.17.008567 19434191

[2] Huang D. Swanson E. A. Lin C. P. Schuman J. S. Stinson W. G. Chang W. Hee M. R. Flotte T. Gregory K. Puliafito C. A. Fujimoto J. G. Swanson E. A., “Optical coherence tomography,” Science 254(5035), 1178–1181 (1991). 10.1126/science.1957169 1957169 PMC4638169

[3] Mariampillai A. Standish B. A. Moriyama E. H. Khurana M. Munce N. R. Leung M. K. Jiang J. Cable A. Wilson B. C. Vitkin I. A. Yang V. X., “Speckle variance detection of microvasculature using swept-source optical coherence tomography,” Opt. Lett. 33(13), 1530–1532 (2008). 10.1364/OL.33.001530 18594688

[4] Izatt J. A. Kulkarni M. D. Yazdanfar S. Barton J. K. Welch A. J., “In vivo bidirectional color Doppler flow imaging of picoliter blood volumes using optical coherence tomography,” Opt. Lett. 22(18), 1439–1441 (1997). 10.1364/OL.22.001439 18188263

[5] Makita S. Hong Y. Yamanari M. Yatagai T. Yasuno Y., “Optical coherence angiography,” Opt. Express 14(17), 7821–7840 (2006). 10.1364/OE.14.007821 19529151

[6] Barton J. Stromski S., “Flow measurement without phase information in optical coherence tomography images,” Opt. Express 13(14), 5234–5239 (2005). 10.1364/OPEX.13.005234 19498514

[7] Morgner U. Drexler W. Kärtner F. X. Li X. D. Pitris C. Ippen E. P. Fujimoto J. G., “Spectroscopic optical coherence tomography,” Opt. Lett. 25(2), 111–113 (2000). 10.1364/OL.25.000111 18059799

[8] Schmitt J., “OCT elastography: imaging microscopic deformation and strain of tissue,” Opt. Express 3(6), 199–211 (1998). 10.1364/OE.3.000199 19384362

[9] de Boer J. F. Milner T. E. van Gemert M. J. C. Nelson J. S., “Two-dimensional birefringence imaging in biological tissue by polarization-sensitive optical coherence tomography,” Opt. Lett. 22(12), 934–936 (1997). 10.1364/OL.22.000934 18185711

[10] Hee M. R. Swanson E. A. Fujimoto J. G. Huang D., “Polarization-sensitive low-coherence reflectometer for birefringence characterization and ranging,” J. Opt. Soc. Am. B 9(6), 903 (1992). 10.1364/JOSAB.9.000903

[11] Everett M. J. Schoenenberger K. Colston B. W. Jr. Da Silva L. B., “Birefringence characterization of biological tissue by use of optical coherence tomography,” Opt. Lett. 23(3), 228–230 (1998). 10.1364/OL.23.000228 18084468

[12] Hitzenberger C. Goetzinger E. Sticker M. Pircher M. Fercher A., “Measurement and imaging of birefringence and optic axis orientation by phase resolved polarization sensitive optical coherence tomography,” Opt. Express 9(13), 780–790 (2001). 10.1364/OE.9.000780 19424315

[13] Pierce M. C. Strasswimmer J. Park B. H. Cense B. De Boer J. F., “Advances in optical coherence tomography imaging for dermatology,” Journal of Investigative Dermatology 123(3), 458–463 (2004). 15304083 10.1111/j.0022-202X.2004.23404.x

[14] Pircher M. Hitzenberger C. K. Schmidt-Erfurth U., “Polarization sensitive optical coherence tomography in the human eye,” Prog. Retin. Eye Res. 30(6), 431–451 (2011). 10.1016/j.preteyeres.2011.06.003 21729763 PMC3205186

[15] Walther J. Golde J. Kirsten L. Tetschke F. Hempel F. Rosenauer T. Hannig C. Koch E., “In vivo imaging of human oral hard and soft tissues by polarization-sensitive optical coherence tomography,” J. Biomed. Opt. 22(12), 1–17 (2017). 10.1117/1.JBO.22.12.121717 29264891

[16] Baumann B., “Polarization Sensitive Optical Coherence Tomography: A Review of Technology and Applications,” Appl. Sci. (Basel) 7(5), 474 (2017). 10.3390/app7050474

[17] Wang Z. Lee H.-C. Ahsen O. O. Lee B. Choi W. Potsaid B. Liu J. Jayaraman V. Cable A. Kraus M. F. Liang K. Hornegger J. Fujimoto J. G., “Depth-encoded all-fiber swept source polarization sensitive OCT,” Biomed. Opt. Express 5(9), 2931–2949 (2014). 10.1364/BOE.5.002931 25401008 PMC4230879

[18] Baumann B. Choi W. Potsaid B. Huang D. Duker J. S. Fujimoto J. G., “Swept source/Fourier domain polarization sensitive optical coherence tomography with a passive polarization delay unit,” Opt. Express 20(9), 10229–10241 (2012). 10.1364/OE.20.010229 22535114 PMC3366588

[19] Lu Z. Matcher S. J., “Absolute fast axis determination using non-polarization-maintaining fiber-based polarization-sensitive optical coherence tomography,” Opt. Lett. 37(11), 1931–1933 (2012). 10.1364/OL.37.001931 22660077

[20] Götzinger E. Baumann B. Pircher M. Hitzenberger C. K., “Polarization maintaining fiber based ultra-high resolution spectral domain polarization sensitive optical coherence tomography,” Opt. Express 17(25), 22704–22717 (2009). 10.1364/OE.17.022704 20052196 PMC2963062

[21] Davé D. P. Akkin T. Milner T. E., “Polarization-maintaining fiber-based optical low-coherence reflectometer for characterization and ranging of birefringence,” Opt. Lett. 28(19), 1775–1777 (2003). 10.1364/OL.28.001775 14514097

[22] Al-Qaisi M. K. Akkin T., “Polarization-sensitive optical coherence tomography based on polarization-maintaining fibers and frequency multiplexing,” Opt. Express 16(17), 13032–13041 (2008). 10.1364/OE.16.013032 18711542

[23] Schoenenberger K. Colston B. W. Maitland D. J. Da Silva L. B. Everett M. J., “Mapping of birefringence and thermal damage in tissue by use of polarization-sensitive optical coherence tomography,” Appl. Opt. 37(25), 6026–6036 (1998). 10.1364/AO.37.006026 18286100

[24] Lu Z. Kasaragod D. Matcher S. J., “Conical scan polarization-sensitive optical coherence tomography,” Biomed. Opt. Express 5(3), 752–762 (2014). 24688811 10.1364/BOE.5.000752 PMC3959841

[25] Byers R., “Clinical Applications of Angiographic Optical Coherence Tomography,” University of Sheffield (2018).

[26] Byers R. A. Matcher S. J., “Ghosting artifact reduction of polarization sensitive optical coherence tomography images through wavelet-FFT filtering,” in *Optical Coherence Tomography and Coherence Domain Optical Methods in Biomedicine XXIII*, Izatt J. A. Fujimoto J. G., eds. (SPIE, 2019), 10867, p. 111.

[27] Huynh-Thu Q. Ghanbari M., “Scope of validity of PSNR in image/video quality assessment,” Electron. Lett. 44(13), 800 (2008). 10.1049/el:20080522

